# The Burden of Dengue in Children by Calculating Spatial Temperature: A Methodological Approach Using Remote Sensing Techniques

**DOI:** 10.3390/ijerph18084230

**Published:** 2021-04-16

**Authors:** Oliver Mendoza-Cano, Pedro Rincón-Avalos, Verity Watson, Abdou Khouakhi, Jesús López-de la Cruz, Angelica Patricia Ruiz-Montero, Cynthia Monique Nava-Garibaldi, Mario Lopez-Rojas, Efrén Murillo-Zamora

**Affiliations:** 1 Facultad de Ingeniería Civil, Universidad de Colima, km. 9 Carretera Colima-Coquimatlán, Col. Jardines del Llano, Coquimatlán 28400, Colima, Mexico; princon0@ucol.mx (P.R.-A.); jlopez71@ucol.mx (J.L.-d.l.C.); ruiz_montero@ucol.mx (A.P.R.-M.); mario_lopez@ucol.mx (M.L.-R.); 2 Health Economics Research Unit, University of Aberdeen, Aberdeen AB25 2ZD, UK; v.watson@abdn.ac.uk; 3 School of Water, Energy and Environment, Centre for Environmental and Agricultural Informatics, Cranfield University, Cranfield MK43 0AL, UK; A.Khouakhi@cranfield.ac.uk; 4 Department of Civil and Environmental Engineering, University of Wisconsin-Madison, 1415 Engineering Dr, Madison, WI 53706, USA; cnava@wisc.edu; 5 Departamento de Epidemiología, Unidad de Medicina Familiar No. 19, Instituto Mexicano del Seguro Social, Av. Javier Mina 301, Col. Centro, Colima 28000, Colima, Mexico

**Keywords:** children, arbovirus, dengue, remote sensing, temperature, health economics

## Abstract

Background: Dengue fever is one of the most important arboviral diseases. Surface temperature versus dengue burden in tropical environments can provide valuable information that can be adapted in future measurements to improve health policies. Methods: A methodological approach using Daymet-V3 provided estimates of daily weather parameters. A Python code developed by us extracted the median temperature from the urban regions of Colima State (207.3 km^2^) in Mexico. JointPoint regression models computed the mean temperature-adjusted average annual percentage of change (AAPC) in disability-adjusted life years (DALY) rates (per 100,000) due to dengue in Colima State among school-aged (5–14 years old) children. Results: Primary outcomes were average temperature in urban areas and cumulative dengue burden in DALYs in the school-aged population. A model from 1990 to 2017 medium surface temperature with DALY rates was performed. The increase in DALYs rate was 64% (95% CI, 44–87%), and it seemed to depend on the 2000–2009 estimates (AAPC = 185%, 95% CI 18–588). Conclusion: From our knowledge, this is the first study to evaluate surface temperature and to model it through an extensive period with health economics calculations in a specific subset of the Latin-American endemic population for dengue epidemics.

## 1. Introduction

Dengue is a mosquito-borne viral infection that has spread throughout the tropical world over the past 60 years and now affects over half the world’s population. The geographical range of dengue is expected to expand due to on-going global phenomena, such as climate change and urbanization [1]. The incidence of dengue fever has increased dramatically worldwide in recent decades; today, 2.5 billion people are at risk of contracting the disease, and Mexico is no exception [2]. Dengue has different stages; this disease is driven by climate factors that the virus must go through in and with the mosquito vector during its transmission. [3,4,5]. Ae. aegypti life cycle is directly influenced by climate changes [6,7,8,9]. Higher temperatures can increase the dengue fever epidemic’s potential by increasing the availability of suitable habitats for mosquitoes to develop and minimize the virus incubation time, thus increasing the transmission rate [3,10]. Climate studies are also relevant from mosquitoes like Anopheles female mosquito (who caused Malaria), while the Aedes aegypti mosquito causes Dengue [11]. Climate change projections onto future conditions showing potential distributional shifts in coming decades similarly lack, at least outside Europe [12]. On the other hand, very high temperatures may also increase mosquito mortality and decrease dengue fever risk [13,14]. Above the optimal temperature, mosquito development rates remain relatively stable and may reduce until temperatures reach an upper limit, at which point development drops dramatically [15,16].

Additionally, rainfall can decrease the effects of dengue fever [13,14]. It is known for people to forget and leave containers outside during the rainy season, which can become suitable breeding grounds for Ae. aegypti [9,14,17,18]. Children are the most vulnerable to those infections [19], mostly in schools because of the epidemic and transmission factors [20]. Furthermore, children over six may be at greater risk of dengue infection [21].

Climate change may affect the virus and the vector, directly and indirectly; this variability can also trigger geographical shifts in the distribution of vector-borne infectious diseases [3]. However, empirical relationships between climate variables, dengue occurrence, and burden have not been firmly established for the Latin American context. Understanding how climate change may influence dengue fever’s burden into new areas and quantifying it with economic measurements is vital to support on-going dengue management strategies and further surveillance and interventions [14,22].

The Institute for Health Metrics and Evaluation (IHME) team presented the Global Burden of Disease (GBD), Injuries, and Risk Factors study in 2016. The GBD is a systematic and scientific effort to quantify morbidity and mortality due to over 300 causes of death and disability [23]. The health economic assessment focused on dengue helps evaluate the impact of certain variables such as the urban temperature and the impact on dengue transmission on children [24,25,26].

For disability and death, time is considered the standard metric; one disability-adjusted life years (DALY) is the equivalent to one year of healthy life lost; in other words, a health gap. The years of life lost due to premature death (YLL) and the years lived with a disability (YLD) in their different degrees of severity are expressed by DALY rates. DALYs are internally consistent and separates comorbidities so that a health loss cannot be ascribed to several causes simultaneously; this avoids double counting [27].

To the best of our knowledge, this is the first study evaluating temperature and a dengue model correlation with remote sensing techniques with an original and specific Phyton code developed and correlated with the disease’s health economic burden in Mexico’s endemic areas. This study evaluates the association between spatial modeled urban temperature and dengue burden from a health financial perspective, using IHME data for 1990–2017. One of the study’s relevant aspects is identifying in the Daymet 3V [28] product a tool to address the problem of inadequate or non-existent information on meteorological parameters in large areas of Mexican territory. This study could be of interest in various studies in which meteorological parameters are involved as covariates in statistical models.

## 2. Materials and Methods

### 2.1. Study Site

**Figure 1:**
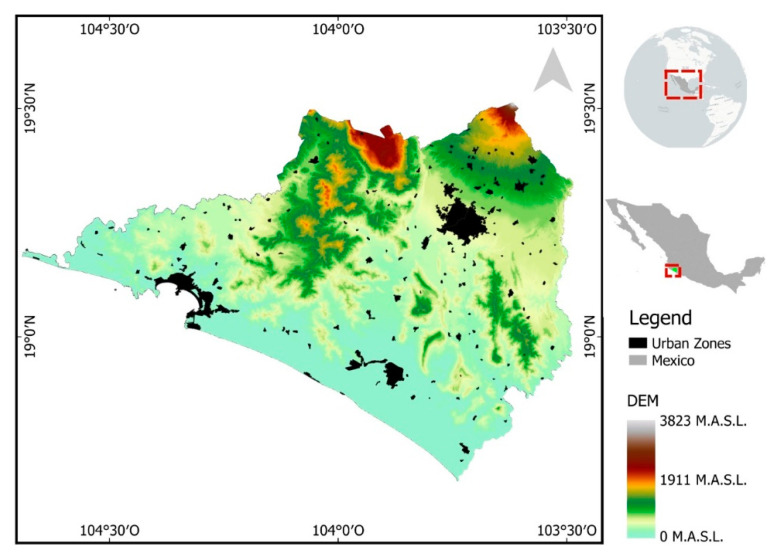
Study site. Colima, Mexico.

Colima is one of the 32 provinces of Mexico and is located in the western part of the country. Colima’s total area is about 5625 km^2^. In terms of climate, the wet season extends from June to November, whereas the dry season is from December to May. Colima has a mountainous landscape in the north, where the Colima Volcano is the highest area, but no urban settlements are near this active mountain (Figure 1).

Colima is divided into ten municipalities, with approximately 711,235 people living in the urban-settlement area of 207.3 km^2^. In Colima state, there are 126 people per square kilometer [29]. In the present study, we used only the urban settlement area as the spatial unit of spatial temperature analysis.

### 2.2. Data Collection and Spatial Analysis

#### Spatial Surface Temperature Collection and Calculation

We select the Daymet V3 dataset [28]: Daily Surface Weather and Climatological Summaries; Daymet V3 provides gridded estimates of daily weather parameters for the United States, Mexico, Canada, Hawaii, and Puerto Rico. The data covers a period from 1 January 1990 to 31 December 2018.

Due the interest in this research for urban temperatures, we used the Daymet database’s gridded information, hosted on the mass storage and processing platform for spatial images, Google Earth Engine [30].

In addition to the Daymet data, we also used station-based temperature data from CONAGUA (National Water Commission in Mexico, Spanish Acronym) stations, distributed throughout the state. Only the stations with more than 80% of daily temperatures per year were used. The station-based data complemented the DAYMET V3 since the stations were very scarce and had low spatial coverage.

Atypical temperature measurements were found during the extraction of temperature data from the Daymet V3 database (generating temperatures close to 50 and 0 degrees Celsius). A statistical filter was used to eliminate dates that presented a behavior above 95 quantiles and below 5 quantiles in both data sources: Daymet and CONAGUA.

### 2.3. Health Economics Burden of Disease of Dengue

#### JointPoint Regression with IHME Metrics

The DALY rates (per 100,000) due to dengue in the state of Colima (Mexico) among school-aged (5–14 years old) children were obtained from the IHME. The data is publicly available and covers a period from 1990 to 2017.

The AAPCs represent a useful epidemiological tool to estimate a population-based parameter, representing the average of the year-to-year change in percentage over the studied period [31]. Ref. [32] JointPoint regression models were employed to compute year-adjusted AAPCs and 95% CI. Furthermore, the overall analysis was performed, and stratified estimates (1990–1999, 2000–2009, and 2010–2017) were also obtained. Moreover, this method has been identified as a valuable tool for making inferences about changes in trends over time [33,34,35,36]. This analytical procedure was performed by using the Stata MP 14.0 (StataCorp, College Station, TX, USA) package.

## 3. Results

### Spatial Surface Temperature Results

**Figure 2:**
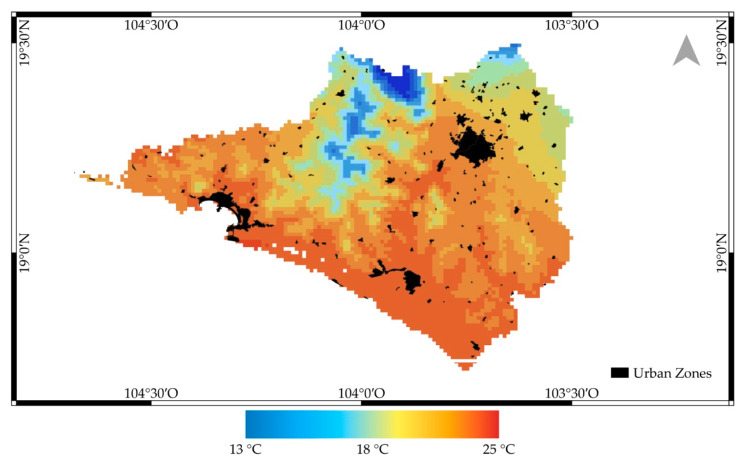
Average annual temperature calculation 1990–2018, Colima Mexico.

**Figure 3:**
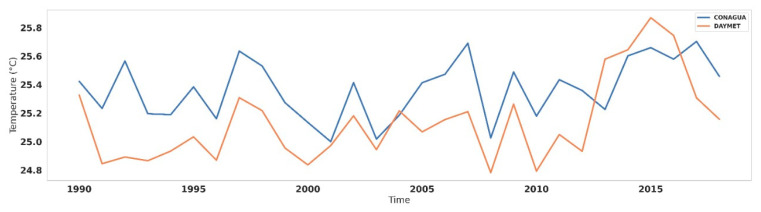
Temperature over time.

The mean average temperature map from Colima 1 January 1990 to 31 December 2018 is shown below in Figure 2. It can be observed that average temperature experiences a gradient between 13 and 25 °C in Colima. A decrease in temperature can be identified towards the north, where the Colima volcano’s foothills are located and to the northwest, where the biosphere reserve of the Sierra de Manantlan is located. It is important to note that the most significant urban centers in the state are located in areas where the average annual temperature is above 23 °C. Figure 3 presents a median temperature over the years of CONAGUA and DAYMET. The temperature data analysis allows us to identify that the DAYMET 3V data can adequately reproduce the behavior of the data recorded at the CONAGUA surface weather stations. An apparent increase can also be observed in the last decade in the average annual temperature. This statistic results from more and more days during the dry season when the maximum daily temperatures are above 30 °C. CONAGUA’s temperature sensors are generally installed at 1.50 m from the surface, so this may be a parameter that affects when compared with DAYMET records generated from information at automatic stations, in which the sensor is located at a higher altitude.

**Figure 4:**
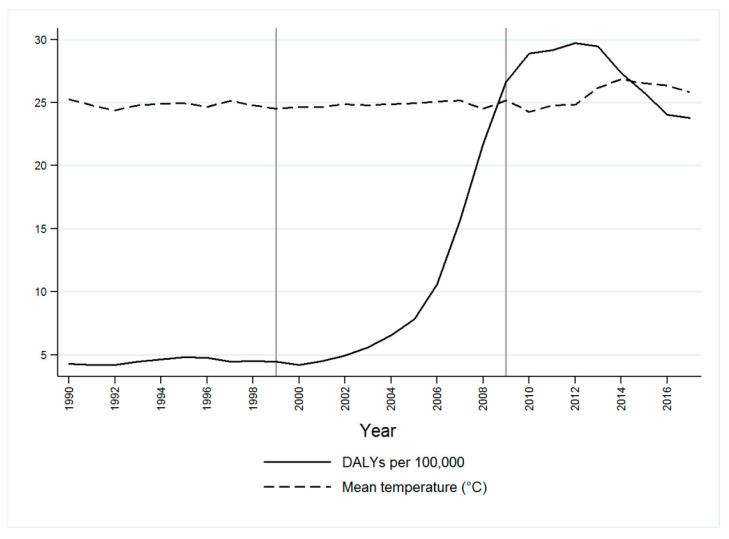
Disability-adjusted life years (DALYs) due to dengue among school-aged children and mean temperatures (°C) in urban areas from Colima, Mexico, 1990–2017.

The yearly dengue-related DALYs rates (per 100,000) during the studied period and the mean temperatures (°C) are presented in Figure 4. We observed that during the second analyzed period (2000–2009), the dengue-burden among school-aged children progressively increased, alongside a slight increase in the mean computed temperatures. A decreasing trend was observed in both estimates from 2014 to 2017.

Table 1 shows the mean temperature and year-adjusted DALYs rates. A positive trend was documented from 1990 to 2017, and dengue rates increased by an average of 64% per year (AAPC = 64%, 95% CI 44–87%). This upward trend seemed to depend on the second analyzed period (2000–2009; AAPC = 185, 95% CI 18–588%). No other estimates were significant.

## 4. Discussion

Our work shows novel calculations in the earth engine platform, with free data and health economic models. We analyzed the association between the mean temperatures in urban areas from a state of Mexico and the dengue-related DALYs among school-aged children during a twenty-seven-year period. Our findings suggest that the dengue burden has increased with mean temperatures, particularly during the second evaluated decade (2000–2009). However, the potential limitations of an ecologic approach have to be considered in interpreting our results.

There are such studies of remote sensing techniques and dengue, for example. Ref. [37] used NDVI, NDWI, LST night, LST day, and TRMM-GPM rain data from 2012 to 2016 as predictive variables and machine learning. Geographic information systems and remote sensing are powerful tools for studying the current distribution and predicting areas of risk for the presence of disease vectors. These tools are excellent for targeting actions for the prevention and control of tropical diseases like dengue, using (ASTER and QuickBird) imagery/Moderate Resolution Imaging Spectroradiometer (MODIS) [38,39,40]. To the best of our knowledge, this is the first study that evaluates surface temperature with remote sensing techniques during an extensive period with health economics calculations in a specific subset of the Latin-American population. Some studies have used operationally available satellite-derived environmental variables (temperature, humidity, and precipitation) for their dengue models [37]. However, we intend to show a new approach using free remote sensing tools to obtain applied health economics models. In an endemic-tropical environment like the study area, the temperature has a direct inference, increasing the availability of suitable habitat for the mosquito to develop and minimize the virus incubation time. As a result of this increase in the transmission rate [3,10,34,41], our findings suggest that spatial surface temperature results can be used with confidence in future scenarios with population mobility related to surface land temperature.

The principal factor contributing to the emergence of the dengue epidemic is urbanization [42]. The study in Taiwan’s urban areas also indicated that risk classification, based on how many months with a monthly temperature being ≥18 °C and level of urbanization, could be a feasible and plausible approach to identifying the regions with a potential risk of dengue fever infection [43].

The projections were difficult to compare because of the different modeling approaches, the variable quality of the data used, and the different variables used to drive disease distribution. The spread, establishment, and persistence of dengue depend not only on temperature but also on characteristics of the natural and human-made environments, rainfall, and travel and trade [14]. In [7], eighty percent of severe dengue cases over 1983–2001 occurred when the temperature was 27–29.5 °C and mean humidity was >75%.

Given that warmer temperatures can bring higher humidity, understanding these interactions is vital for early warning systems and projecting how a changing climate could alter the dengue’s future burden. A changing climate may also affect dengue’s geographic range and incidence through effects on human and natural systems, such as water storage, land use, and irrigation. We modeled the mean temperature-adjusted dengue burden in Mexico’s western state among school-aged children (aged 5–14 years old), and an upward trend was documented. Therefore, we provide statistical evidence of an association between rising environmental temperatures and the vector-borne disease-related burden in these age-specific subjects. According to previous analyses, school-aged children were chosen for the study group [20]. Climate change, travel, migration, and global trade are the primary cause of transmission of diseases such as chikungunya fever, dengue fever, and other emerging infectious diseases in places like Europe [44]. Every 1 °C increase in monthly average temperature increases the total population at risk for dengue fever by 1.95 times [43]. With the increasing temperature trends, regions that are now not suitable for dengue might be soon. Such regions could invest in health economic evaluations and mitigation policies of dengue impacted regions. This investment could produce significant health cobenefits by providing resources to the affected region and gathering evidence on how to direct future investigations should the new area become affected.

When it comes to determining the risk of dengue occurring in a given region, the extrinsic incubation period (EIP) plays an important role. The EIP is commonly defined as “the interval between the acquisition of an infectious agent by a vector and the vector’s ability to transmit the agent to other susceptible vertebrate hosts” [45]. For example, for dengue, once a mosquito has ingested the virus through a blood meal, the virus will spread through the mosquito’s body, escape the midgut, and ultimately reach the salivary glands (SG). The SG is where the virus can be passed on to another host during the next blood meal [46]. Most of the models implemented for dengue use fixed values for the duration of the EIP or rather rough estimates of temperature dependence [47]; therefore, this manuscript’s methodology is useful to use EIP models in future studies of temperature dependence. The relationship found in this study helps to shed light on the link between weather and dengue for the development of future dengue prediction models while vaccines are not available.

Under the empirical temperature relationships considered in [48], they found that *r* (epidemic growth rate) peaked at a temperature threshold robust to uncertainty in model parameters that do not depend on temperature. However, this temperature threshold’s precise value could be refined following future studies of empirical temperature relationships. Although *r* will vary across different regions for different reasons, the finding on [49] that temperature changes under future climate change could increase the epidemic intensity of dengue in some areas suggest a categorically new way climate change might impact infectious disease transmission.

On the other hand, mosquito Anopheles population abundance is more sensitive to temperature than previously thought. It is strongly influenced by the dynamics of the juvenile mosquito stages; whose vital rates are also temperature-dependent [50]; this implies that this presented study and approach can help study malaria and other tropical diseases in climate change conditions on other parts of the globe.

The framework for identifying such temperature thresholds offers a new way to classify regions where dengue virus epidemic intensity could increase or decrease under future climate change and underlines the need for more methodologic studies to show the burden of various infectious diseases.

## 5. Conclusions

Here we used a range of 1990–2017 medium surface temperature and dengue prevalence data in Colima, Mexico, to model temperature-based relationships in a burden transmission model. We employed remote sensing techniques to assess the correlation between spatial temperatures and dengue burden, high in Mexico, where the study was conducted. Our results suggest the hypothetical negative impact of upward trends of mean temperatures and, according to our findings, partially determining increasing trends in dengue burden among the analyzed population. Our results are useful for other researchers to make more and better models using free-data accessible using Daymet-V3, especially for those who develop models of extrinsic and intrinsic incubation periods (EIP and IIP). The results obtained in this study open the door to future studies. Some other vector-borne infectious diseases such as West Nile Virus, Lyme disease, and malaria also can be modeled if relevant data is available.

The DAYMET V3 product demonstrated its potential for use in Mexico and other Latin American countries, where the monitoring of meteorological parameters still relies on insufficient measurement networks. Other variables such as rainfall and evaporation are incorporated, which, like temperature, can present a significant correlation that allows multiple linear regression models to be generated using meteorological parameters as covariates. An important aspect is to study the variables on a monthly scale, allowing us to identify time lags in the correlation between the variables.

However, the complexity of dengue-determinants must be considered as well as the limitation of an ecological approach.

## Figures and Tables

**Table 1:** Annual Percent Change of DALYs rates. Abbreviations: DALYs, Disability-adjusted life years; APC, the annual percentage of change.

Period	AAPC	(95% CI)	*p*
Overall (1990–2017)	64	(44, 87)	<0.001
First (1990–1999)	2	(−68, 227)	0.976
Second (2000–2009)	185	(18, 588)	0.020
Third (2010–2017)	−5	(−18, 10)	0.503

Note: The showed AAPCs were adjusted by year and the mean annual temperature and were computed through Jointpoint regression models. (Full model, 14.6%, First period, 1.1%, Second period, 6.4%, Third period, 1.0%).

## Data Availability

The authors encourage collaboration and use of the data by other re- searchers. Data are stored on the server in Mexico, and researchers interested in using the data for scientific purposes should contact the project leader Oliver Mendoza-Cano.
